# Exploring the use and perceived impact of artificial intelligence in medical internship: a cross-sectional study of Palestinian doctors

**DOI:** 10.3389/frai.2025.1738782

**Published:** 2025-12-10

**Authors:** Abdallah Qawasmeh, Salahaldeen Deeb, Alhareth M. Amro, Khaled Alhashlamon, Ibrahim Althaher, Nour Yaser Mohammad Shadeed, Khadija Mohammad, Farid K. Abu Shama

**Affiliations:** 1 Health Education and Scientific Research Unit, Minstry of Health, Ramallah, Palestine; 2 Faculty of Medicine, Al-Quds University, Jerusalem, Palestine; 3 Faculty of Graduate Studies, An-Najah National University, Nablus, Palestine

**Keywords:** academic performance, artificial intelligence, clinical competence, internship doctors, medical education, time management

## Abstract

**Background:**

Artificial intelligence (AI) is increasingly used in medical education to support academic learning, clinical competence, and efficiency. However, the extent and impact of AI usage among medical interns, particularly in Palestine, remain underexplored.

**Objective:**

This study aimed to assess the prevalence of AI usage among internship doctors in Palestine and evaluate its perceived impact on their academic performance, clinical competence, time management, and research skills.

**Methods:**

A cross-sectional survey was conducted with 307 internship doctors in Palestine. The survey collected data on the frequency and types of AI tools used, including ChatGPT, and interns’ perceptions of AI’s impact on their training. Demographic information, such as age, gender, and university affiliation, was also gathered to explore potential associations with AI usage patterns.

**Results:**

The study found that 76.9% of interns used AI regularly, with ChatGPT being the most popular tool (76.2%). Despite frequent use, only 3.3% reported formal AI training. The majority of interns perceived AI as beneficial in improving academic performance (61%), clinical competence (67%), and time management (74%). Notably, time management showed the highest perceived improvement. However, 75.9% expressed concerns about becoming overly reliant on AI, fearing it could diminish critical thinking and clinical judgment. Age and university affiliation were associated with differences in AI usage patterns and perceived benefits, with older interns and those from international universities reporting greater perceived improvements.

**Conclusion:**

This cross-sectional study highlights the widespread use of AI among internship doctors in Palestine and generally positive perceptions of its educational value, particularly for academic performance and clinical competence. However, it also reveals a substantial gap in formal AI training, suggesting a need for structured, ethically grounded AI education in medical curricula. Because the study is exploratory and cross-sectional, these findings should be interpreted as perceived associations rather than evidence that AI use or training causes improved outcomes; future longitudinal and interventional studies are needed to clarify long term effects.

## Introduction

Artificial intelligence (AI) is reshaping how physicians learn and work, with rapid advances now touching admissions, instruction, assessment, and clinical reasoning across medical education (Gordon et al., 2024). While early evidence suggests that AI can augment traditional teaching and facilitate self-directed learning, the educational literature also emphasizes the need to align tools with sound pedagogy and outcome-focused evaluation rather than novelty alone (Gordon et al., 2024). Parallel calls from medical educators highlight that curricular integration has lagged behind technological progress, leaving many learners to experiment with AI informally and without clear guidance on competencies, ethics, or assessment (Lee et al., 2021).

The emergence of large language models (LLMs) has accelerated this trend. Systems such as ChatGPT have demonstrated performance at or near passing thresholds on high-stakes examinations like the United States Medical Licensing Examination, renewing debate about how generative AI might support knowledge acquisition, reasoning, and feedback in medical training (Kung et al., 2023). These capabilities create tangible opportunities for just-in-time explanation, writing support, and exam preparation yet they also risk overreliance, superficial learning, and the propagation of model errors if learners are not trained to appraise outputs critically.

Global policy work underscores these dualities. The World Health Organization’s guidance on the ethics and governance of AI for health stresses data protection, transparency, accountability, equity, and human oversight as prerequisites for responsible use in clinical and educational settings (World Health Organization, 2021). For learners, this translates into curricular needs that go beyond technical literacy to include bias recognition, privacy stewardship, and safe-use practices that preserve clinical judgment and professional identity.

Evidence from the Middle East similarly points to rapid uptake coupled with uneven preparation. National surveys report high exposure to AI tools among medical students in Saudi Arabia but limited formal instruction and only moderate “AI readiness,” reinforcing the gap between widespread use and structured training (Al Shahrani et al., 2024). Yet most published work centers on undergraduate learners; little is known about how AI is actually used during the internship year a pivotal transition from student to physician when time pressure, clinical responsibility, and identity formation intensify.

Although AI usage in medical education has been extensively studied across Europe, the Gulf, and East Asia, recent meta-analyses show a complete absence of data from conflict-affected, low-resource, or politically fragmented health systems. These contexts differ fundamentally from well-resourced environments due to digital infrastructure limitations, variable curriculum integration, and inconsistent access to AI tools. To date, no published study has examined how these constraints shape AI adoption among interns in Palestine—a population undergoing the critical transition from student to independent clinician. Therefore, this study provides a novel contribution by offering context-specific evidence from a uniquely under-represented health system, adding essential regional variation to the global discussion on AI readiness in medical education.

This study addresses that gap by examining internship doctors in Palestine. Using a cross-sectional survey, we quantify the prevalence and patterns of AI use, characterize perceived educational and clinical impacts (academic performance, clinical competence, research skills, and time management), and test associations with demographics, training, and university background. We also describe perceived challenges and risks, including infrastructural barriers and concerns about dependency. By grounding discussion in a national cohort of interns, the study aims to inform locally relevant curricula, faculty development, and governance policies that promote beneficial, safe, and equitable use of AI in early postgraduate training.

## Methodology

### Study design and setting

We conducted a cross-sectional survey between June and September 2025 to explore the use, perceptions, and impact of artificial intelligence (AI) tools among internship doctors in Palestine. The study targeted interns affiliated with the Palestinian Ministry of Health (MoH). A cross-sectional design was selected because it allows for the assessment of behaviors and perceptions within a defined population at a single point in time. The study was exploratory and descriptive, and all analyses were designed to identify patterns of association rather than to infer causality. Data were collected electronically using a structured, self-administered questionnaire hosted on Google Forms. The survey link was disseminated through MoH training centers, professional WhatsApp groups, and official social-media channels.

A stratified convenience sampling strategy was used, meaning that the internship population was first divided into predefined subgroups (strata) and then interns who were available and willing to participate were recruited by convenience within each subgroup, to ensure representation across major training hospitals and internship rotations. Interns were recruited from all Ministry of Health teaching facilities in the Hebron district. In this study, the strata were defined by training hospital (each Ministry of Health teaching facility in the Hebron district) and by major internship rotations (e.g., internal medicine, surgery, pediatrics, obstetrics and gynecology, and other core rotations), reflecting the actual distribution of interns across these settings. Although this non-probability approach was necessary due to logistical constraints, aligning recruitment with these hospital- and rotation-based strata helped reduce over-representation of any single site or specialty and increased the representativeness of the sample in relation to the internship population.

### Participants

Eligible participants were internship doctors aged ≥18 years who were actively enrolled in the Ministry of Health internship program and had access to AI-based tools. No exclusion criteria were applied on the basis of previous AI exposure or formal AI training. Participation was entirely voluntary, and all responses were collected anonymously. The study population was estimated at approximately 1,500 interns. Assuming an expected prevalence of AI use of 50%, a 5% absolute margin of error, and a 95% confidence level, the minimum required sample size was calculated using the single-proportion formula with finite population correction for *N* = 1,500, yielding a target sample of 306 interns.

A total of 412 invitations were distributed, of which 307 interns completed the survey, yielding a response rate of 74.5%. To minimize selection bias, invitations were disseminated across multiple channels including WhatsApp academic groups, Ministry of Health training centers, email notifications, and in-person reminders during orientation sessions. No incentives were provided. The gender and university distribution of respondents were compared with national internship statistics and showed no significant deviation, suggesting minimal risk of selection bias.

### Questionnaire development and structure

The questionnaire was adapted from several previously validated surveys that investigated AI usage and AI readiness among medical students (Yousef et al., 2025; Hanifa et al., 2025; Sumner et al., 2025) and was modified for the internship context. These instruments were selected because they assessed constructs central to our research questions, including AI awareness, frequency and purposes of use, perceived educational benefits, and concerns about over-reliance. Item wording was adjusted to reflect internship roles (for example, replacing ‘medical students’ with ‘internship doctors’ and adding internship-specific tasks), while preserving the original constructs. Items clearly irrelevant to the Palestinian internship context were omitted to reduce respondent burden. The adapted questionnaire underwent expert review by medical educators and AI researchers to ensure content validity and contextual appropriateness.

The final instrument contained five sections:

*Demographics:* age, gender, university, internship duration, and previous AI training.

*AI use patterns:* frequency of use, preferred platforms (e.g., ChatGPT, research tools), and main purposes (research, study support, clinical preparation).

*Perceived benefits:* Likert-scale items assessing AI’s contribution to academic performance, clinical competence, time management, and research productivity.

*Internship experience:* evaluation of major rotations (internal medicine, pediatrics, surgery, obstetrics and gynecology, psychiatry, emergency medicine, and public health) and structured courses (trauma care, infection control, ethics, professionalism, and advanced research methods).

*Professional reflections:* questions on how internship experiences influenced specialty choice and the transition from student to practicing physician.

Completion time averaged 8–10 min. The full questionnaire, including the five sections, response scales, and the grouping of items into perception subscales, is provided in Supplementary File 1, which also includes sample items for each domain.

### Validation and pilot testing

The initial questionnaire was reviewed by three domain experts for clarity, face validity, and internal consistency. Minor linguistic and structural revisions were made to optimize comprehensibility and alignment with internship-specific experiences. A pilot test among 20 interns confirmed clarity and flow; their data were excluded from the final analysis.

### Data collection procedure

The survey remained open from June 25 to September 25, 2025. Participants were required to provide electronic informed consent before proceeding. Data were collected directly through Google Forms and exported into a secure database. Incomplete or duplicate responses were excluded during data cleaning.

### Data management and statistical analysis

Data were analyzed using RStudio (R version 4.x). Descriptive statistics summarized participant demographics and AI usage. Categorical variables (e.g., gender, university affiliation, AI training, frequency of use) were reported as frequencies and percentages; continuous variables (e.g., age, Likert-scale means) as mean ± standard deviation.

For inferential analysis, associations between categorical variables were assessed using the chi-square test. Independent t-tests compared mean scores between two groups (e.g., gender, AI training), while one-way ANOVA tested differences across more than two categories (e.g., universities, age groups). Post-hoc tests identified significant pairwise differences where applicable. Pearson’s correlation was used to examine relationships between continuous variables such as age, frequency of AI use, and perceived benefits across academic, clinical, and research domains. Internal consistency of the multi-item Likert scales (perceived academic benefit, clinical competence, time-management impact, and research skills) was assessed using Cronbach’s alpha. A value of ≥0.70 was considered acceptable and indicative of adequate internal reliability. Statistical significance was defined as *p* < 0.05. Given the exploratory, cross-sectional nature of the study, *p*-values were interpreted descriptively to highlight patterns of association rather than as evidence of causal relationships.

### Ethical approval

The study protocol was reviewed and approved by the Al-Quds University Institutional Review Board (ref. no. 592/REC/2025). The research complied with the principles of the Declaration of Helsinki. Participation was voluntary, and anonymity and confidentiality were strictly maintained. Only the research team had access to the dataset.

## Results

The internal consistency of the four perception scales demonstrated acceptable reliability, with Cronbach’s *α* = 0.81 for academic performance, 0.84 for clinical competence, 0.78 for time-management impact, and 0.83 for research skills.

### AI usage patterns

**Figure 1:**
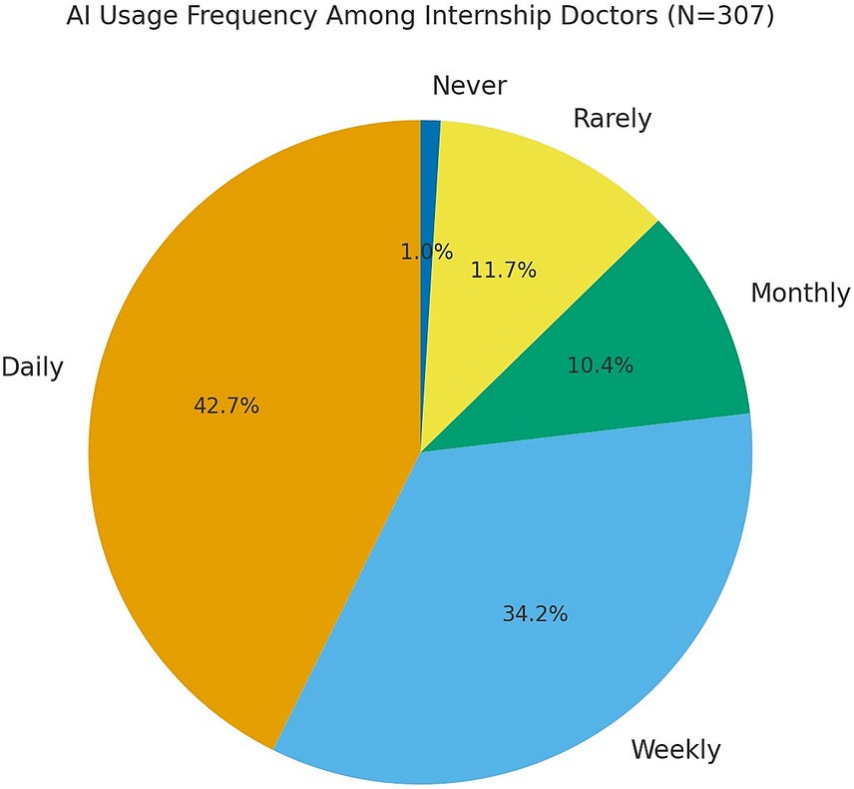
Distribution of AI application usage frequency among internship doctors (*N* = 307).

A total of 307 internship doctors in Palestine completed the survey (mean age 25.4 ± 1.4 years; range 21–36; 51.5% male) as shown in Table 1, with 96.7% in their 6th year. AI use was routine: 76.9% reported daily or weekly use (daily 42.7%, weekly 34.2%) as shown in Figure 1; the next most common category was “rarely” (11.7%). ChatGPT was the most-used application. Only 3.3% had formal AI training, indicating predominantly informal learning. Usage did not differ by gender (χ^2^ *p* > 0.30). Interns with training showed higher daily use than untrained peers (70% vs. 42%), but this was not statistically significant (χ^2^ *p* ≈ 0.46). By contrast, usage frequency differed by age (χ^2^ *p* = 0.047): interns >25 years were more likely to report daily use (47% vs. 41% among ≤25 years), and “never use” occurred only in the older group (3 of 83 vs. 0 of 222). Overall, AI tools were nearly universally adopted and used routinely in this cohort.

**Table 1:** Presents key demographic characteristics and AI usage patterns of the participants.

Variable	Category	*N*	%
Gender	Male	158	51.50%
	Female	149	48.50%
Medical program duration	6 years	297	96.70%
	5 years	10	3.30%
University region	Out-Palestine	193	62.90%
	In-Palestine	114	37.10%
Age (years)	Mean ± SD	25.4 ± 1.4	—
AI training	Yes	10	3.30%
	No	297	96.70%
AI use frequency	Daily	131	42.70%
	Weekly	105	34.20%
	Monthly	32	10.40%
	Rarely	36	11.70%
	Never	3	1.00%
Most used AI application	ChatGPT	234	76.20%
	Research tools	60	19.50%
	others	4	1.30%

### Perceived impact of AI on education

**Figure 2:**
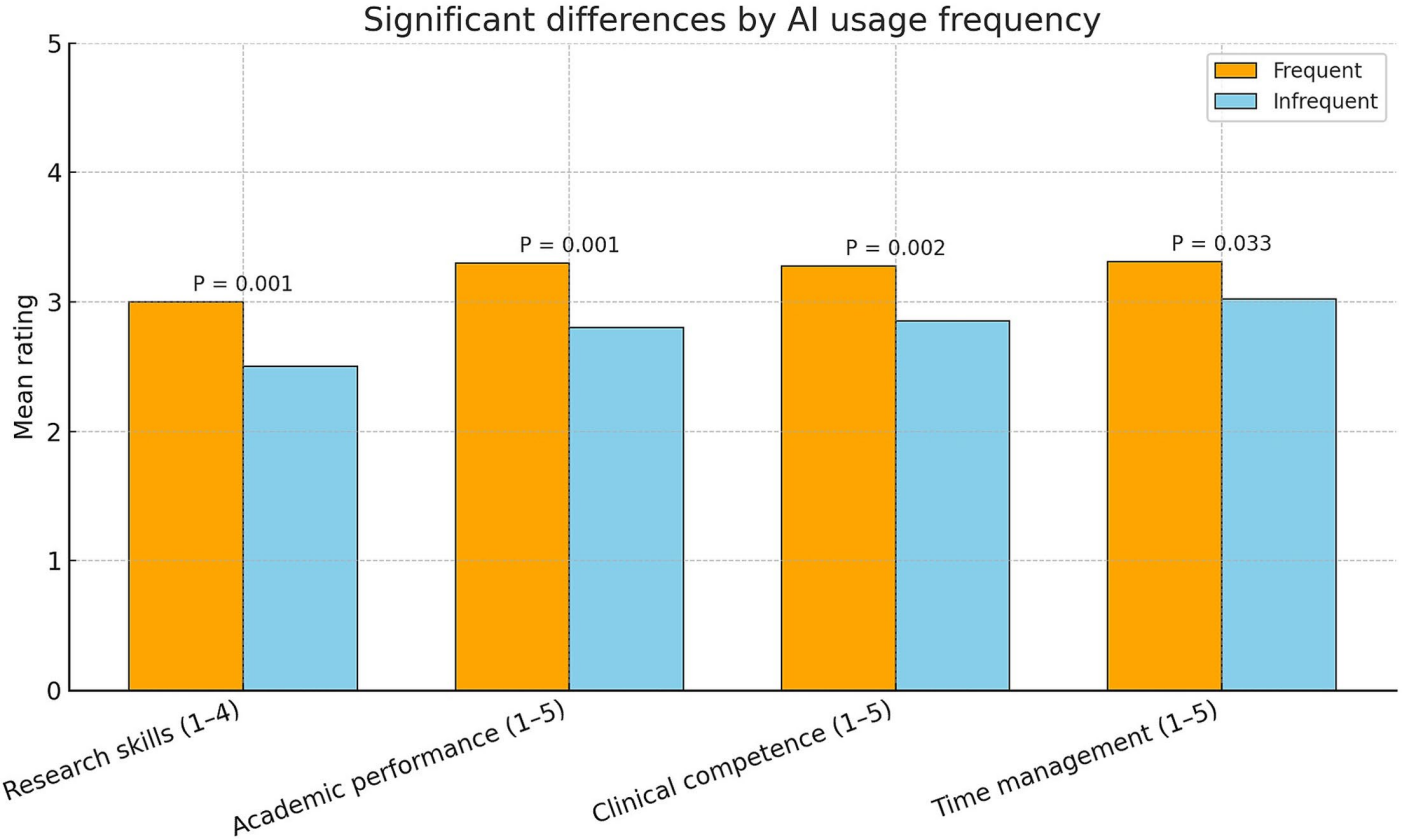
Differences in perceived outcomes by AI usage frequency. Bars show mean self-ratings for four outcomes among internship doctors (*N* = 307), comparing Frequent AI users (daily/weekly; orange) with Infrequent users (monthly/rarely/never; blue). Scales: research skills (1–4), academic performance (1–5), clinical competence (1–5), and time management (1–5); higher scores indicate greater perceived improvement/effectiveness. *p* values from two-sample comparisons between frequency groups are annotated above bars.

Interns reported broadly positive educational impacts from AI. For academic performance, 61% perceived improvement (31% unsure, 8% no improvement); the mean self-rating was 3.18 ± 0.92 on a 5-point scale (median = 3, “moderate”). Perceived enhancement of clinical competence was likewise moderate (mean 3.18 ± 0.93), with responses concentrated in “moderate positive” (67%) and “strong positive” (17%); 15% reported no impact and 1% a negative impact. Time management received the highest perceived benefit (mean 3.23 ± 1.05): 74% reported at least a moderate improvement (including 20% “greatly”), whereas 5% rated AI “not at all” effective. Research skills also showed perceived gains, with 54% reporting moderate enhancement and 19% significant enhancement (4% no improvement). Overall, the most frequently endorsed benefits were at least moderate improvement in time management (74%), moderate clinical impact (67%), and improved academic performance (61%) as shown in Figure 2, indicating consistent, moderate advantages across academic, clinical, and efficiency domains.

### Associations with demographic factors

We examined whether age, gender, university, and AI-training status were associated with AI usage and perceived outcomes. Significant associations involved age, training, and university; gender showed none. Older interns (>25 years) reported greater perceived benefits: improved academic performance as in Table 2 (72% vs. 57% among ≤25; χ^2^ *p* = 0.015) and higher mean ratings (academic 3.31 vs. 3.13; clinical 3.39 vs. 3.10), with the clinical difference significant (*p* = 0.003). Age correlated positively with improvement scores (academic r = 0.12, *p* = 0.038; clinical r = 0.17, *p* = 0.0035) but not with time-management ratings (*p* = 0.34). Usage patterns also differed by age (ANOVA *p* = 0.033): older interns were somewhat more often daily users and accounted for all “never” users.

**Table 2:** Highlights four key significant associations between demographic factors and AI-related outcomes.

Demographic comparison	Outcome metric	Group 1 vs. Group 2 (value)	*p*-value
Age > 25 vs. ≤ 25 years	% reporting improved academic performance (yes)	72.3% vs. 56.7%	0.015*
Age > 25 vs. ≤ 25 years	Mean clinical competence improvement score (1–5)	3.39 vs. 3.10	0.003*
Formal AI training vs. no training	% reporting “significantly” enhanced research skills	60% vs. 18%	0.004 *
Gender (male vs. female)	% daily AI usage	43% vs. 42%	0.85
Gender (male vs. female)	Mean academic performance improvement score (1–5)	3.23 vs. 3.11	0.25

*p-value significant at less than 0.05.

Formal AI training, although rare, was associated with higher perceived research-skill gains: 60% of trained interns vs. 18% of untrained rated the impact ‘significant’ (χ^2^ *p* = 0.004). Trained interns also reported higher mean academic (3.50 vs. 3.16) and clinical (3.60 vs. 3.16) ratings, though these differences were not statistically significant, likely reflecting the small trained sub-sample (*n* = 10). Usage frequency did not differ by training status.

University location in (Palestine vs. out) also mattered. Compared with major local universities (*n* = 210), interns from smaller or foreign programs (*n* = 97) reported higher academic improvement (mean 3.34 vs. 3.10; *p* = 0.045) and a trend toward higher clinical competence (3.33 vs. 3.10; *p* = 0.066), with similar overall usage rates. By contrast, gender showed no significant differences in usage or perceived benefits (e.g., academic improvement 65% males vs. 56% females; *p* = 0.14; all 1–5 impact ratings *p* > 0.25).

Participants reported that the internship year had a substantial perceived impact on their development. About three-quarters (72.6%) reported that it helped them choose a future specialty (χ^2^ *p* < 0.0001). The perceived influence on personality and professional identity was even stronger, with 88.3% stating that the internship shaped their transition from student to physician (χ^2^ *p* < 0.0001). These findings suggest that, in interns’ self-reports, the internship year is an important period for specialty consideration and professional identity formation; however, the cross-sectional design precludes causal inference.

Focusing on AI usage frequency and university location, frequent users generally reported higher training/benefit ratings across specialties, but this difference reached statistical significance only in Internal Medicine (frequent 3.88 vs. non-frequent 3.39; *p* = 0.009). In Obstetrics & Gynecology, Pediatrics, General Surgery, Emergency Medicine, Public Health, and Mental Health, frequent vs. non-frequent differences were not significant (*p* = 0.536, 0.244, 0.792, 0.185, 0.120, and 0.126, respectively). By university background, ratings were broadly similar except in Obstetrics & Gynecology, where graduates educated outside Palestine reported higher scores than those educated within Palestine (3.41 vs. 2.91; *p* = 0.038). No other specialty showed a university effect (Pediatrics *p* = 0.737; General Surgery *p* = 0.575; Internal Medicine *p* = 0.983; Emergency Medicine *p* = 0.931; Public Health *p* = 0.985; Mental Health *p* = 0.414) as shown in Table 3.

**Table 3:** Evaluation of the specialties and skill acquisition by it: differences by gender, AI usage frequency, and university location (Palestine vs. outside).

Specialty	Male mean	Female mean	*p* (gender)	Frequent mean	Non-freq mean	*p* (freq)	In-pal mean	Out-pal mean	*p* (univ)
Obstetrics & gynecology	3.25	3.5	**0.044**	3.39	3.3	0.536	2.91	3.41	**0.038**
Pediatrics	3.93	3.74	0.124	3.88	3.7	0.244	3.76	3.85	0.737
General surgery	3.85	3.52	**0.012**	3.7	3.66	0.792	3.52	3.7	0.575
Internal medicine	3.94	3.59	**0.012**	3.88	3.39	**0.009**	3.76	3.77	0.983
Emergency medicine	4.45	3.88	**<0.001**	4.22	4.01	0.185	4.19	4.17	0.931
Public health	3.57	3.19	**0.008**	3.45	3.17	0.12	3.38	3.39	0.985
Mental health	3.06	2.83	0.1	3.01	2.74	0.126	3.14	2.93	0.414

Bold values are significant at less than 0.05.

Regarding challenges and risks associated with AI use, only a minority of students reported direct difficulties: 16% had encountered challenges in applying AI to academic or clinical work. In contrast, a majority (61.9%) experienced technical barriers or access issues, highlighting infrastructural limitations as the main obstacle rather than resistance to AI itself. Importantly, nearly three-quarters of respondents (75.9%) expressed concern that students may become over-reliant on AI tools for learning and research. These findings suggest that while practical hurdles and risks of dependency are widely recognized, relatively few students perceive AI itself as inherently problematic in their training.

To further explore independent associations with AI-related outcomes, a multivariable linear regression model was constructed adjusting for age, gender, university location (inside vs. outside Palestine), AI-training status, and frequency of AI use. After adjustment, more frequent AI use was independently associated with higher self-rated improvement in academic performance (*β* = 0.27, *p* = 0.004) and clinical competence (β = 0.31, *p* = 0.002). AI training was independently associated with higher perceived enhancement of research skills (*β* = 0.42, *p* = 0.001), whereas age showed a modest but statistically significant association with clinical-competence ratings (β = 0.15, *p* = 0.036). Gender was not a significant predictor in any model. University background outside Palestine was associated with slightly higher academic-performance ratings (β = 0.18, *p* = 0.041), but was not related to time-management scores. These relationships are associative and based on self-report rather than evidence that AI use or training causes better performance.

## Discussion

This study provides one of the first examinations of AI usage among internship doctors in a resource-constrained and politically fragmented healthcare system. Unlike studies conducted in high-income or technologically advanced institutions, the findings highlight that infrastructural limitations, inconsistent curricular integration, and variable access to digital tools coexist with patterns of AI use and perceived readiness in Palestine, although the cross-sectional design does not allow directionality to be determined. This contextual contribution fills a critical gap in the global literature and introduces perspectives from an under-represented region.

Recent systematic reviews and meta-analyses consistently report high interest but limited preparedness among medical learners globally, especially in low- and middle-income countries. However, none of these syntheses include data from Palestine or comparable conflict-affected contexts. The present study therefore expands the evidence base by offering region-specific insights into AI adoption during the internship year.

Consistent with our quantitative findings, most interns reported frequent use of AI tools, particularly ChatGPT, and perceived moderate educational benefits across academic, clinical, and time-management domains.

Demographic factors such as age and university affiliation were associated with differences in AI usage and perceived outcomes. In our sample, older interns tended to report more frequent AI use and somewhat higher clinical-competence ratings than younger interns, and graduates of international universities reported slightly higher academic-competence ratings than those trained locally. These patterns may reflect greater exposure to AI-related content or more confidence in experimenting with new technologies among some subgroups, but they remain associative and should be interpreted cautiously. At the same time, many interns expressed concerns that excessive dependence on AI could impair their ability to think critically and make independent clinical decisions. This concern is particularly relevant in settings where clinical reasoning, contextual judgement, and ethical decision-making are central to safe practice. Together, these findings underline the importance of integrating AI in ways that support and enhance core educational experiences rather than substituting for them.

The findings have important implications for national policy and curriculum planning. Structured AI training should be integrated into undergraduate and internship curricula, supported by unified national standards outlining core AI competencies. Policymakers should prioritize investments in digital infrastructure and faculty development to ensure equitable access to AI tools across all training hospitals. Such reforms may help align Palestinian medical training with international digital-health standards and reduce gaps between local and internationally trained graduates.

Further evidence from Yousef et al.’s study in Palestine underscores similar challenges in understanding the practical implications of AI in clinical settings (Yousef et al., 2025). While the students in Yousef et al.’s study reported significant improvements in academic performance and research productivity due to AI, clinical competence was perceived to have benefited only moderately. This pattern was consistent with our findings, where interns reported more substantial improvements in time management and academic performance, but the impact on clinical competence was less pronounced. These results highlight the ongoing challenge of integrating AI into clinical training, where practical applications of AI tools are often limited by the lack of direct clinical exposure and hands-on use of AI for patient care.

The findings from Hanifa et al. (2025) align with our study in highlighting the widespread use of AI tools among Palestinian medical students and interns (Hanifa et al., 2025; Yousef et al., 2025). Both studies found high levels of AI awareness, with 83% of students in Hanifa et al.’s study and 96.7% of interns in our study reporting frequent AI use. However, Hanifa et al. observed lower scores in AI ethics and vision, pointing to gaps in understanding the broader implications of AI, a trend reflected in our study where clinical competence was less impacted by AI. Both studies also found that prior AI experience and academic performance were positively correlated with AI readiness, reinforcing the need for more comprehensive AI education that includes ethical and strategic considerations, alongside technical skills, to better prepare students for the challenges of AI in healthcare. As a result, the findings of this study are consistent with and build upon existing literature on the use of AI in medical education. While AI has been reported to offer perceived benefits for academic performance, clinical competence, and time management in multiple studies, the lack of formal training and concerns about over-reliance highlight critical gaps that need to be addressed. This study emphasizes the need for further integration of AI into medical curricula and the importance of fostering a balanced approach to ensure that AI enhances, rather than undermines, essential clinical skills.

The perceived benefits and risks observed in this study have important implications for medical education. Interns reported that AI tools are particularly helpful for time management and academic support, a pattern consistent with studies from Saudi Arabia and Palestinian medical students that describe stronger perceived gains in academic and research domains than in clinical competence (Al Shahrani et al., 2024; Yousef et al., 2025; Jebreen et al., 2024; Hanifa et al., 2025). At the same time, only a very small minority reported any formal AI training, and many expressed concern about over-reliance and the potential erosion of critical thinking (Yousef et al., 2025; Jebreen et al., 2024; Rony et al., 2025; Khakpaki, 2025). These findings suggest that AI is already embedded in learners’ daily routines, but its use is largely informal and unguided (Yousef et al., 2025; Jebreen et al., 2024). Rather than discouraging AI use, curricula should provide structured, ethically grounded training that teaches students to appraise AI outputs critically, recognize limitations and potential biases, and use these tools as a complement not a substitute for foundational knowledge, clinical reasoning, and professional identity formation (Rony et al., 2025; Khakpaki, 2025).

In addition to formal training, medical institutions should also consider the infrastructural challenges highlighted by this study. The technical barriers and access issues reported by 61.9% of interns underscore the need for improved access to AI tools and reliable digital infrastructure in medical training centers. Ensuring that all students have equitable access to AI resources will be key to maximizing the benefits of AI integration across diverse medical institutions, especially those with limited resources (Borges do Nascimento et al., 2023). Finally, the differences observed in AI usage and perceived benefits based on age and university background suggest that tailored approaches may be necessary to meet the needs of different groups of interns. Older interns, for example, may benefit from more targeted AI training that acknowledges their prior educational experiences, while interns from international universities may require support in adapting to local medical training environments (Tortella et al., 2025). These variations should be considered when designing AI training programs to ensure that all interns, regardless of their demographic background, can fully benefit from AI tools.

The multivariable analysis provides further insight into factors associated with AI-related benefits during internship. Even after controlling for demographic and educational variables, frequent AI use remained independently associated with higher academic and clinical-competence ratings, suggesting that more active engagement with AI tools may coincide with more positive perceptions of their impact. The association between formal AI training and higher research-skill ratings points toward a potential role for structured instruction, although the very small number of trained interns limits firm conclusions. Additionally, the modest but statistically significant association between age and clinical-competence ratings may reflect differences in maturity or prior clinical exposure. Overall, these regression findings suggest that AI readiness is linked not only to individual motivation but also to training opportunities, curricular structure, and institutional context.

Beyond descriptive trends, this study also points to multilevel correlates of AI readiness, including prior exposure, perceived utility, and institutional digital infrastructure. These contextual factors suggest that AI adoption is influenced not only by individual preference but also by broader structural and educational conditions. This aligns with emerging competency-based frameworks that emphasize ethical, contextual, and practical readiness over unstructured adoption.

This study has several notable strengths, particularly its large sample size of 307 internship doctors, which strengthens the reliability and generalizability of its findings. By focusing on the internship year, the study provides valuable insights into a crucial phase in medical training, when interns transition from students to practicing physicians. The inclusion of demographic variables such as age, gender, and university background adds depth to the analysis, highlighting how these factors influence AI usage and its perceived benefits. This comprehensive approach allows for a more nuanced understanding of how AI is being utilized across different subgroups of interns, offering practical insights that can inform future curriculum development and interventions aimed at optimizing AI integration in medical education.

This study has several limitations. First, its single-region sampling frame (Hebron) limits external validity, especially given regional differences in digital access and training opportunities. Second, the cross-sectional design prevents causal inference regarding the relationship between AI usage and perceived educational benefits. Third, findings rely on self-reported perceptions, making them vulnerable to recall and social-desirability biases. Fourth, the very small number of interns with formal AI training limits meaningful comparison between trained and untrained groups. Future research should include multi-center sampling, longitudinal designs, validated measurement instruments, and objective performance-based outcomes.

Future research should address the limitations of this study by utilizing a longitudinal design to track the long-term effects of AI tools on medical education and clinical performance. Additionally, given the minimal formal AI training reported, future studies could explore the impact of structured AI education on interns’ learning outcomes, clinical decision-making, and critical thinking skills. Further research should also investigate the specific AI tools used by interns and their effectiveness in enhancing different aspects of medical training, such as research skills, clinical reasoning, and time management. Finally, exploring the ethical implications of AI use in medical education such as the potential for bias in AI algorithms and the ethical considerations of AI-assisted decision-making will be crucial in ensuring responsible AI integration into curricula and clinical practice.

## Conclusion

This study provides context-specific evidence on AI usage among internship doctors in Palestine, demonstrating widespread adoption but minimal structured preparation. AI tools were perceived to enhance academic performance, clinical competence, and time management, yet concerns regarding over-reliance emphasize the need for responsible and supervised integration. These findings have direct implications for national curriculum reform, capacity-building, and responsible AI governance in medical education. Future multi-center longitudinal studies incorporating objective outcome measures and validated instruments are needed to guide the safe and effective integration of AI into early postgraduate training.

## Data Availability

The original contributions presented in the study are included in the article/supplementary material, further inquiries can be directed to the corresponding author.
